# Spontaneous Cure of Phthisis

**Published:** 1844-11

**Authors:** 


*Spontaneous cure of Phthisis.*—Dr, Clarkson, in the New York Journal of Medicine, reports the following case of spontaneous cure of consumption:

It is my belief that there are many more cases of phthis pulmonalis cured by nature alone than medical men are generally aware of. I do not mean to say that we should always trust to nature alone, by any means, but that we should be very cautious lest we do too much. The case that I am about to relate came under my care the 29th day of March last. Sarah Hamor, æt. 55, born in England, had always been rather delicate. She was attacked last fall with erysipelas of the left hand and arm; and after recovering from that, she caught a severe cold, which was followed by a troublesome cough, and shooting pains through the chest, but more especially on the right side, from the mamma to the shoulder blade of the same side. She now began to lose flesh; and when I first saw her (which was as above stated) she was so much emaciated, to use a common phrase, that she was a mere skeleton. She was so weak, at that time, that she was unable to help herself; she had profuse night sweats, severe cough, and expectorated about half a pint or more of light greyish yellow pus of a frothy nature (same as usually seen in the last stages of consumption) in the course of twenty-four hours. Her shoulders were raised and brought forward; the chest flat, and the clavicles very prominent, leaving a hollow. On auscultation there was that peculiar hollow gurgling sound, at times, heard over the right lung; and, at others, there was no sound to be heard of respiration at some points. The dyspnœa sometimes became so urgent that the friends several times thought her dying. Almost as soon as she fell asleep, she would break out in a profuse perspiration, and the little sleep she did get was not refreshing. There was an abscess on the right side of the spine, extending from about the second rib to the ninth or tenth, and about four inches wide, involving all the muscles of the back contained within that space. There was an opening about an inch and a half from the spine, over the region between the fifth and sixth ribs, which I enlarged by means of a bistoury, and kept open by the use of poultices. The abscess discharged constantly, and at one time nearly a pint of well marked pus. When I was first called, I told the friends that I thought she could live but a very short time, which conclusion, I was informed, all parties concerned had come to before having her brought into town

She had been brought over the river from Wallabout, L. I., the day that I first saw her—it being her desire to be at her friend's house, in this city, when her spirit should take its flight. My great object in her case was to palliate her sufferings, and merely make her as comfortable as possible during the time she might live. I told her friends to let her eat as much as she wanted, and anything in the way of ordinary food; also to let her have a small quantity of wine. Ordered the following mixture to be given at night, and at any time during the day when the cough was most troublesome: R. Gum Acacia, Ext. Glycyrrhiz. ä ä ℥j., Syr. Althea. Tinct. Opii. ä ä ℥ ss., Vin. Antimo. gtt. x. and Aqua pura 3 iii. Dose, cochlea magn. I visited my patient daily, expecting every visit to be the last, for about a fortnight, when I was convinced that she was no worse: her night sweats began to to decrease, and she got more refreshing sleep; her appetite seemed very good, and in fact was rather voracious, and her cough was easier. The discharge from her back began to diminish, and at the end of four weeks was quite healed up. Her cough had nearly left her at that time, and she began to gain flesh. In about four weeks from the time I first saw her, cough had entirely left her, and she was gaining strength fast. The only thing that she now complained of was pain about the right side, and a "falling in" as she said, of the right side of the chest, which she had observed during the whole course of her treatment. The right side became much smaller than the left, owing to the chest's shaping itself to the new condition of the lung. In about six weeks from the time I first saw her she left town for Poughkeepsie, in better health, she said, than she had enjoyed for years. I gave her nothing of any importance beside cough mixtures, similar to the one first ordered.

There is something very singular in this case, and some may be led to believe that there were no tubercles in the lungs, the affirmative of which I have every reason to believe, for I never saw a plainer marked case. The left lung seemed, from the first, not to be diseased.

